# The Dental Register of the West

**Published:** 1848-01

**Authors:** 


ART. V.—*The Dental Register of the West.* Vol. I., No. I. Cincinnati. 1847.

The progress of society in this great interior of the continent, is indicated by signs indefinitely numerous. One of them is its medical journals. The first ever published, was in Cincinnati, in the year 1822 and '23. It was edited by the lamented and indefatigable Dr. Godman, and based on a subscription list previously procured, but not used, by one of the editors of this Journal. It continued till six quarterly numbers were issued. Since that time, (to say nothing of those, which, like the one just mentioned, lived for a greater or less period, and then ceased to be,) we have the following, now in circulation, which we shall designate by the towns in which they are published—Montreal, Buffalo, Chicago, St. Louis two, New Orleans, Memphis, Louisville, and Cincinnati—in all nine. To these we may now add the *quasi* medical magazine, of which the title is placed at the head of this article. Its appearance is under the auspices, or rather by order of the "*Mississippi Valley Association of Dental Surgeons*," whose annual meetings are held in Cincinnati. Its editors are a committee of that body, composed of Dr. James Taylor, of that city, and Dr. B. B. Brown, of St. Louis, both practical dentists. Its publication is to be quarterly—its extent 200 pages *per*

*annum*—its price two dollars. The first number, for October last, is now before us, and having just finished a hasty perusal of it, we proceed to give such a notice of its contents, as will serve to make its objects and character known to our readers; many of whom, we trust, will encourage it with their names, both as subscribers and contributors. In expressing this hope, we do it in the full conviction, that every physician should have a better acquaintance with the diseases of the teeth and gums, than he is likely to acquire from the systematic works of the profession; and in the further conviction, that the periodical before us, if adequately patronized, will give him the information needed.

The first article, consists of the proceedings of the late annual meeting of the Association, at Cincinnati, in the second week of September; with an appended address from the president, Dr. E. Taylor, of that city, containing much excellent advice on the means of elevating the profession of the dental surgeon.

To this succeeds a paper, read at the late meeting of the same body, by Dr. Berry, of the same city, on the effects upon the teeth and gums, of the habitual use of tobacco. The conclusion to which the author comes, (and that correctly, we believe,) is not merely adverse to its preservative power, but unfavorable to its use. The following paragraphs seem to us so worthy of dissemination, that we are tempted to extract them:

"On the leaves of tobacco when gathered, there is always some dust from the soil that is not entirely removed in the process of manufacturing, which, by its mechanical action, abrades the teeth in chewing the cud. And if the dropping of water wears away stones, will not the constant grinding of tobacco, under the presure of the masseter muscles, wear away the teeth?

"I have seen several cases of tobacco chewers, who, before they had lived half of the three score and ten, had worn the teeth on which they masticated their cud, so as to make the corresponding sides of their faces shorter than the other sides. They assured me that they could not quit the use of tobacco, and that to chew it on the teeth unaccustomed to it, produced nausea. In one of these cases considerable deformity was caused by the wearing away of the front teeth of one side; while those of the other remained of full length.

"The narcotic and nauseating properties of tobacco, may remove the pain of an aching tooth, for which it is frequently prescribed, instead of the forceps. A gentleman of my acquaintance, being directed by his physician to chew tobacco for the toothache, continued the practice for thirty years, when upon mature deliberation he concluded he had chewed long enough for a pain that never occurred but once, and sagely resolved to abandon the filthy habit. He did so, but not without experiencing considerable suffering for several days.

"The opinion that chewing tobacco preserves the teeth from decay is, I think, erroneous. It arises, probably, from the fact that many persons find their teeth to decay less rapidly after commencing its use than before; but they resort to it at a time when their teeth contain a larger proportion of lime than at an earlier period, and are therefore less liable to be affected with caries. And, besides, people generally take better care of their teeth, after they arrive at the age in which they usually commence chewing tobacco than previously to that time, preventing in a great degree the causes of their decay.

"The pressure of the cud on the gums of the molares and bicuspides, not unfrequently causes them to recede, leaving the roots of these teeth exposed and liable to decay; while the gums are in many cases inflamed, and seldom in a perfectly healthy condition.

"The smoking of tobacco injures the teeth by the sudden changes of temperature, to which it exposes them It renders the mouth most disgustingly filthy, coating the inner surfaces of the teeth with black empyreumatic oil, exhaling an odor like that of a much used tobacco pipe, and inducing diseased action in the gums.

"In some sections of our country rubbing snuff on the teeth and gums, called dipping, is in common and popular use, especially among the ladies. It is appled with little swabs or brushes made by chewing the ends of peices of soft wood. The pretended reason for dipping—and no one could indulge in so filthy and disgusting a habit without some excuse for it—is that it preserves the teeth from decay. A "social dip" requires some half a dozen participants, each with a brush, which they all dip into one bottle of snuff and rub their teeth and gums for a considerable length of time; while the stimulus of the snuff adds excitement to the conversation. By this practice the snuff is frequently impacted in the interstices of the teeth, and insinuated beneath the edges of the gums, which is sometimes so extensive in its effects as to cause a loosening of the teeth. From several years observation of the teeth of a large number of persons who use snuff in this manner, I am induced to believe, that it has no tendency to preserve the teeth from decay; and that they are more likely to be attacked with caries from the general diseased state of the gums."

The next article is is by Dr. Slack, Professor of Chemistry in the Ohio College of Dental Surgery, and embraces an argument, on chemical principles, against the use of metallic amalgams for filling decayed teeth, the gist of which is, that they generate a galvanic action, which promotes a decay of that part of the teeth with which they are in contact, concerning which there can be no doubt.

The article which follows, is an extract from a paper, read at the meeting referred to, by Mr. Allen, of Cincinnati, on the restoration of the contour of the face, after the loss of teeth. To this end he has invented different pieces of gold apparatus, some of which we have seen, and the effects of which, in reference to the object in view, are quite satisfactory and even striking.

Then follows a paper by the same gentleman, on destroying the nerves of decayed teeth, in which he states that, in most cases, the result is unsatisfactory.

The succeeding paper, by Dr. E. Taylor, of the same city, is on the mechanical labor of filling teeth, which we shall not analyze.

To this, succeeds a letter from Mr. H. R. Smith, of Terre Haute, Indiana, on inflammation of the gums and mucous membranes of the mouth, accompanied with morbid sensibility of the teeth, for the successful treatment of which he has, in a number of cases, found the antiphlogistic method indispensable.

The next in order, is an introductory lecture delivered, in 1846, at the opening of the College of Dental Surgery, by Dr.

Rogers, one of the oldest practitioners of Cincinnati. Its subject is 'nerve killing,' and the result of his ample experience is decidedly against the practice.

Then follows a letter from Dr. Joseph Sanford, of Chillicothe, Ohio, giving a short account of a *third* dentition. His patient was a woman 82 years old, who in her 60th year had lost her last tooth.

An account of this phenomenon, bearing some analogy to the return of vision in old age, is contained in the following extract:

"Ten teeth in all have made their appearance. Two lower incisors, two upper front incisors, four bicuspides, above right and left, two lower bicuspides left side. These teeth, with the one exception, are small pieces of brown bone when cut, and act as foreign bodies, which become very painful to the patient, and have to be extracted as fast as cut. One which was perfectly formed was very white when first extracted, but has since turned brown."

The last article which we shall notice, and the last of much importance in the number before us, is on the letheon gas, so intimately connected (in its application) with dental operations. A letter to the editors, from Dr. Joseph Taylor, of Maysville, in this State, informs us that he has used it in about 350 cases. Of the whole, the following is, he says, the most favorable case:

"A lady of sanguine temperament called on me a few months since, for the purpose of having a tooth extracted under the influence of the vapor, requesting me to give her as little as possible to produce the desired effect. Accordingly, after inhaling a very small portion, she observed that she felt its influence. I then separated the gum from one side of the tooth, and inquired if she felt any pain. She answered in the negative; and after the gum was entirely separated, I interrogated her again; her answer was still the same, observing that I must be in haste, that she felt it passing off. I immediately removed the tooth, and, to her great delight, without any suffering whatever."

Another case stands in such strong contrast with this, that we are tempted to extract it. The patient was a gentleman of a high nervo-bilious temperament, to whom Dr. T. administered it with reluctance:

"After inhaling it for abont forty seconds, his breathing became long, full and easy, and in a few moments his face became somewhat florid, the pupil of his eye dilated, and exhibited some agitation, when with one stretch of his arm, he sent me, letheon and all, some distance across the room. I immediately presented myself to him again, and with some difficulty succeeded in removing the tooth, causing much suffering, which continued some time after the effects of the letheon passed off; he was unconscious of every thing except the pain consequent upon the operation, which he described as being excruciating."

Another case still, is worthy of being read, from the fact that the patient was not only far advanced in life, but labored under a chronic pulmonary affection:

"About five months ago I called on a lady about sixty-eight years of age, who had been laboring under pulmonary disease for several years, for the purpose of extracting a tooth. She requested me to put her under the influenee of the vapor; I hesitated, but as it was the direction of her physician, I immediately proceeded to perform the operation, which was done without giving her any pain whatever. The vapor appeared to have salutary effects. I had forgotten to name that she had been confined to her bed for several months. She rested the following night better than usual. The next morning she requested me to give it to her again, as its influence was so very pleasant, which I did with the same success, and all passed off without any bad effects upon her system."

As the experience of Dr. Taylor, in the use of the letheon, has been extensive, and he appears to be an accurate observer, we shall venture to republish one case more, not only because of its peculiarities, but on account of the general remarks which accompany it and the great importance of the subject which it contributes to illustrate:

"A gentleman who inhaled it, and upon whom it had the most happy effect, causing the most thrilling sensation of pleasure, submitted cheerfully to an operation which was entirely unknown to him until he awoke from his reverie, when he assured me that he did not know that I was present. A day or two afterwards I administered it to him again, producing the same pleasant sensation, but an unwillingness to have me operate; since when he has inhaled it two or three times, always producing the same symptoms, yet evading me when I attempted to approach. Each time it had less effect upon him; the last time it passed off almost as soon as he ceased inhaling it. Others again have taken it seven or eight times, always with success; yet I could perceive the more frequently it was inhaled, the more it took to produced the desired effect, and would pass off more rapidly. It is but seldom that a person of a very excitable nervous temperament can be brought sufficiently under its influence so as to prevent all pain consequent upon a severe operation, though it may mitigate it, and in some instances it would be admissible to give it to such. Yet if we do not succeed by the first effort, it is but seldom that a repetition will produce the desired result."

To Dr. Taylor's paper there is appended a letter from Dr. T. McGarrough, of Greenfield, Ohio, giving an account of the amputation of a breast while the patient was under the influence of the letheon. She felt not the least pain, and was entirely free from any subsequent shock or depression.

In concluding this part of our bibliographical notice, it affords us pleasure to be authorized to say, on the authority of Stockton's Dental Intelligencer, that Dr. Morton has quit selling patent rights for his discovery of the use of letheon, or volatilized ether, in surgical operations. Of the propriety of granting the patent many doubts might be raised. Sir Humphrey Davy had long before suggested the inhalation of nitrous oxide, for the mitigation of pain; more than 50 years ago the inhalation of sulphuric ether was proposed by Dr. Richard Pearson for curative purposes;* and fifteen or twenty years since, the inhalation of sulphuric ether, as a substitute for nitrous oxide, was a prevalent amusement with the boys in our streets, many of whom were injured by it. If every

*Breathwaite's Retrospect, Part XV.

new application of a well known therapeutic agent were patented, the world would soon be filled with monopolies. If Drs. Morton and Jackson had been the inventors of sulphuric ether, or the first to propose its inhalation, their claim to governmental protection would have been very different. But in making these remarks, we must not be understood, as attempting to detract from their merits; for, on the contrary, we desire to award to them the highest praise; and, while we do not believe etherization always an efficient aid to the surgeon, nor free from bad effects to every patient, we heartily tender to its authors an expression of gratitude.

Having presented our readers with a tolerably full analysis of the first number of a journal devoted to one of the specialities of the profession, and designed to improve its character, we cannot lay down the pen, without expressing our strong approbation of the enterprize, and our earnest hope for its success. Hitherto, the medical profession have been signally at fault, in reference to diseases of the teeth. We have, in fact, been strangely inconsistent; for while we have utterly neglected to inform ourselves, and, thereby, compelled the people to seek assistance out of the profession, we have united in deriding the uneducated dental mechanics, who have rashly undertaken to supply our delinquencies. We have cherished the lithotomist, the aurist, and the oculist, acknowledged them as professional brethren, deferred to their experience, and promoted their practice; but the dentist has been placed and kept under our ban. He has been first made an empiric, and then treated with contempt. All this is most absurd, for the diseases of the teeth belong as legitimately to the profession, as the diseases of the heart or the eye; and, in many cases, require as much sound judgment and practical adroitness for their cure. With these convictions we hail, with great satisfaction, every attempt to promote their study, and elevate the dignity of those who devote themselves to dental surgery. Under this feeling, we have noticed with regret, the very imperfect manner in which the first number of this new periodical has been brought out. Its errors of orthography are, on the whole, more numerous and glaring than those of any medical work which has hitherto fallen under our eye. The editors seem to be, to some extent, aware of this; and offer as apologies, the short time allowed to bring out the number, and the necessary absence of the one, whose duty it was to superintend the press. These causes of typographical inaccuracy should, however, have been obviated; and, for the sake of the work itself, we hope to see its future numbers exempt from such blemishes.

